# Semirigid thoracoscopy: an effective method for diagnosing pleural malignancies

**DOI:** 10.2478/raon-2013-0068

**Published:** 2014-01-22

**Authors:** Ales Rozman, Luka Camlek, Izidor Kern, Mateja Marc Malovrh

**Affiliations:** 1 Endoscopy and Pulmology Department, University Clinic Golnik, Slovenia; 2 Intensive Care Department, University Clinic Golnik, Slovenia; 3 Pathology Department, University Clinic Golnik, Slovenia; 4 Pulmology Department, University Clinic Golnik, Slovenia

**Keywords:** flex-rigid pleuroscopy, pleural biopsy, pleural effusion, safety, thoracoscopy

## Abstract

**Background:**

Thoracoscopy with a semirigid instrument is a recent technique for diagnosing pleural diseases. The purpose of this study was to report diagnostic yield and complications of the method.

**Patients and methods.:**

Patients with pleural effusion of unknown origin and/or pleural irregularities suspicious for pleural malignancy were included after less invasive means of diagnosis had failed. All procedures were performed under local anaesthesia with intravenous sedation/analgesia with a single point of entry with a semirigid thoracoscope (Olympus LTF-160). Data were collected prospectively between 2008 and 2012.

**Results:**

One hundred fifteen thoracoscopies were performed on 111 patients. The median age was 65 years (range 28–86 years), 14.4% were female and 85.6% male. Seventy-three (65.8%) patients had malignant pleural disease (malignant mesothelioma, metastatic cancer) and 38 (34.2%) had benign disease. The sensitivity, negative predictive value, and accuracy of the procedure for malignancy were 96.0%, 93.0%, and 97.4% respectively. Pleurodesis was carried out in 34 patients; in 32 (94.1%) it was assessed as successful after 1 month. There were 24 adverse events: three empyemas/pleural infections, three bronchopleural fistulae after chest tube placement and lung re-expansion, five patients had excessive pain after pleurodesis, six patients had sedation-associated hypotension, and seven patients had self-limited fever after plerodesis. One patient died 11 days after a procedure for advanced carcinoma.

**Conclusions:**

Semirigid thoracoscopy is an accurate and safe method for evaluation of pleural diseases and useful for therapeutic talc pleurodesis.

## Introduction

Pleural effusion and pleural irregularities are frequent causes for referral for diagnostic evaluation.^1^–^3^ However, obtaining an accurate diagnosis sometimes represents a considerable challenge, even though changes in the pleural space are radiologically obvious.^3^,^4^ Approximately 25% of pleural abnormalities remain unexplained after repeated thoracenthesis and/or closed pleural biopsies.^1^–^3^

The role of medical thoracoscopy is well established in the diagnostic process for the pleural cavity, although the majority of published data on diagnostic yield and safety have been obtained from studies with rigid instruments.^1^–^3^,^5^ Rigid thoracoscopy has certain advantages, including a spacious and readily available entrance to the pleural cavity, which enables good sampling and several therapeutic approaches, in which adhesiolysis and talc poudrage are most widely used.^6^ However, the angle of view is limited and a second entry point is sometimes needed for thorough inspection and sampling. It also requires sufficient pleural space for insertion, which should be at least 10 cm in depth and 10 cm in length or width, which could represent an obstacle in the case of thick adhesions.^7^ The second entry channel can be an additional risk for tumour seeding in cases of malignancy.^8^

The semirigid thoracoscope was recently developed and initial experiences with it show that the method is easy to use and could overcome some of the disadvantages of rigid thoracoscopy.^9^–^11^ The initial published experiences have proven the new technique to be safe, well-tolerated, and effective in evaluating pleural effusion of unknown origin.^9^,^11^–^14^ The angle of view is extended in all directions because of the flexible tip and offers a better overview of the pleural cavity. However, the main concern is the flexible forceps, which lack mechanical power in the sampling process and delay the entire procedure.^10^,^15^

Studies with new semirigid instruments are therefore needed to define their value. The purpose of our report is to contribute additional data about the diagnostic accuracy and safety of semirigid thoracoscopy in the diagnosis of unilateral pleural effusion with a focus on predictive values for pleural malignancy.

## Patients and methods

### Patients

The study period was carried out between 2008 and 2012 with follow-up until February 2013. The patients included were at least 18 years old with unilateral pleural effusion of unknown origin and/or pleural irregularities suspicious for pleural malignancy, and they were candidates for thoracoscopy after less invasive means of diagnosis had failed. We excluded patients with uncontrolled bleeding tendency, unstable cardiovascular status or severe heart failure, ECOG performance status 4, and persistent hypoxemia after evacuation of pleural fluid.

The procedure was explained to patients verbally and in writing, and signed informed consent was obtained before the procedure. The data collection for purpose of this study was approved by the Institutional Ethics Committee.

The study was conducted at a single centre, prospectively by a predefined set of data to be collected.

### Procedure technique

Semirigid thoracoscopy was performed using an Olympus LTF-160 autoclavable thoracoscope (Olympus Tokyo, Japan). We obtained biopsy samples using flexible FB-55CD-1 Olympus forceps with a cusp outer diameter of 2.4 mm and length of 3.5 mm.

The procedures were performed in the endoscopy suite through the single point of entry technique. Patients were placed in the lateral decubitus position with the affected side of the thorax upwards. Continuous visual monitoring and measurements of blood pressure, pulse rate, and haemoglobin saturation were performed. Topical anaesthesia was achieved using 2% lidocaine. Patients received intravenous fentanyl and midazolam for analgesia and sedation. Supplemental oxygen was given through a nasal catheter.

All patients had a chest CT-scan before the procedure and chest ultrasound was performed on the spot for selection of the entry site. Pneumothorax was introduced under C-arc fluoroscopic control and the thoracoscope was typically inserted through selected intercostal space. All pleural fluid was removed after insertion and the pleural space was thoroughly inspected, biopsies were taken, and talc plurodesis performed where indicated (4 g of sterilized talc, Novatech, France). Pleurodesis was avoided in patients with an inconclusive macroscopic appearance or with signs of lung entrapment. A 24F chest tube was placed at the end of the procedure and a chest radiograph was obtained afterwards.

### Histopathology techniques

Biopsy specimens were immediately fixed in 4% neutral buffered formalin. Paraffin embedded tissue sections were HE stained and evaluated. In the case of neoplastic morphology, immunohistochemistry was performed using various antibodies on an automated platform (Benchmark XT, Ventana, Tucson, Arizona).

### Follow up

Non-specific pleuritis was accepted as the final diagnosis after 12 months of follow-up when no other definitive diagnosis was made during that time.

### Data analysis

Descriptive statistical methods were used for data analysis (range, mean, standard deviation).

## Results

One hundred twenty-three patients were referred for semirigid thoracoscopy during the study period. In 12 (9.8%) of them we were unable to induce pneumothorax because of fibrothorax or extensive adhesions. Data were evaluated from 115 procedures on 111 patients; one patient had thoracoscopy on both sides on two occasions and in three patients thoracoscopy was repeated on the same side. The clinical and demographic data of the patients included are presented in (Table 1).

The mean follow-up was 22.3 (± 12.1) months after the procedure.

Seventy-three (65.8%) patients had malignant pleural disease and 38 (34.2%) benign disease (Table 2). Most of the pleural malignancies were malignant mesotheliomas, followed by secondary carcinomas with origins in the lung (*n* = 13), breast (*n* = 3), head and neck (*n* = 3), or rectum (*n* = 1), or of unknown origin (*n* = 4). Benign diagnoses included non-specific pleuritis, asbestos pleuritis, tuberculous pleuritis, rheumatoid pleurisy, and one case of hemothorax. The patient with hemothorax had no history of physical trauma, but received anticoagulant treatment at the time of presentation. Overall diagnostic accuracy, sensitivity, and positive and negative predictive value for malignancy were 97.4%, 96.0%, 100%, and 93.0% respectively.

Five patients had clinical presentations that did not entirely match with the diagnostic findings from thoracoscopy. One patient underwent a surgical diagnostic procedure in which the diagnosis of non-specific pleuritis was confirmed. Three patients had repeated thoracoscopy at clinical deterioration several months later. Two of them were diagnosed with mesothelioma on a second occasion and one still had non-specific pleuritis, which was the final diagnosis after follow-up. One patient with suspicion for malignant mesothelioma had severe comorbidity and was treated conservatively thereafter. Diagnosis of malignant mesothelioma was confirmed at autopsy after his death.

Talc pleurodesis was carried out in 34 patients with macroscopically obvious malignant pleural disease and without evidence of trapped lung during the same procedure. Thirty-two of them (94.1%) were assessed as successful after 1 month.

Six serious adverse events were recorded in four patients. There were three serious adverse events associated with infection. The first patient presented with fever, malaise, and chest pain 1 week after the procedure with pleurodesis. Empyema caused by methicillin-sensitive *Staphylococcus aureus* (MSSA) was found. The patient recovered after chest-tube drainage and antibiotic treatment. In another two patients the signs of pleural infection were associated with trapped lung, subsequent bronchopleural fistula, and prolonged chest drainage (up to 22 days). No pathogens were isolated from pleural space or other samples (hemocultures, urine, sputum) and both patients recovered within a few days of antibiotic treatment. In the fourth patient there was prolonged air leak due to bronchopleural fistula, extensive subcutaneous emphysema, and prolonged chest-tube drainage, without signs of infection.

Among the minor adverse events were seven cases of transient self-limited fever (38ºC or more). Additional analgesia was required in five patients postoperatively after talc poudrage. Six patients had a transient hypotensive period associated with sedation during the procedure, which was reversed by application of plasma-expander.

Seventeen patients (five malignant mesothelioma, four secondary carcinoma, eight non-specific pleuritis) had trapped lungs and re-expansion was not achieved by chest tube drainage immediately after the procedure. Three cases were complicated by bronchopleural fistula, mentioned above.

One patient died 11 days after a procedure for advanced carcinoma.

## Discussion

Rigid thoracoscopy has been a valuable method for evaluating and treating unilateral pleural effusion for more than a century.^16^,^17^ It is used to avoid surgical exploration of the pleural space after cytological examination of pleural fluid and/or after closed needle biopsy of pleura has failed to retrieve diagnostic material.^5^ The new, semirigid instrument has already proved its safety and accuracy in phase II studies, but the level of evidence is still limited in comparison to rigid thoracoscopy.^9^,^11^,^12^ In a previous pilot study we evaluated both techniques for diagnostic value and found comparable diagnostic yields.^15^ Although the biopsy specimens were smaller in the semirigid technique arm, the quality of the samples, interpretability, and immunohistochemistry staining allowed accurate pathological diagnoses. With that experience, we assume that a large multicentric randomized study including hundreds of patients would be required to establish the non-inferiority of the semirigid technique with sufficient power.

Our series of 115 semirigid thoracoscopies is the largest analysed and reported so far. We have confirmed the high diagnostic yield, which is comparable to our previous data from historic cohorts obtained with a rigid instrument and which was between 91.5% and 94%.^16^,^18^,^19^ The majority of patients referred had pleural malignancy: either malignant mesothelioma or metastatic cancer. In this study the prevalence of malignancy was 65.8% compared to 55.2% and 62.4% from our previously published data.^18^,^19^ The high percentage of malignant mesothelioma patients in comparison to patients with secondary carcinoma in our sample is a reflection of the inherent low diagnostic yield of less invasive diagnostic techniques for diagnosing malignant mesothelioma and their low ability to establish a reliable subtype diagnosis and stage, which decisively influences the treatment and prognosis of malignant mesothelioma patients.^7^,^16^,^20^–^22^

Non-vascularized pleural adhesions and/or loculations that could impede the range of vision and pleural fluid removal were present in a large percentage of our patients. We were able to use the semirigid instrument for fenestration and removal of the fluid and thus achieve a wider range of inspection and favourable conditions for successful pleurodesis. An additional contribution to the wide range of visibility in the pleural cavity is the flexible tip, which enables inspection of the pleural area around the entry point and better maneuverability in partly septated pleural space without establishing a secondary entry port.

Procedures were well tolerated and patients did not report pain at the site of the insertion. There were no premature terminations of the procedure because of pain, intolerance, or hemodynamic instability. Large volumes of pleural fluid were safely aspirated, but some patients experienced coughing and chest discomfort after lung re-expansion with a chest tube. However, there were no cases of re-expansion pulmonary oedema. Additional intravenous anaesthesia was required in five patients after pleurodesis. All cases of serious adverse events underwent detailed analysis. Patients with pleural infection recovered after chest-tube drainage and antibiotic treatment. Trapped lung was regarded as a characteristic of the existing pleural disease, which was complicated by prolonged air leak in three patients.

The role of thoracoscopy for patients with undiagnosed unilateral pleural effusion is therefore important for several reasons.^23^ It has a high diagnostic yield and good safety profile, and is well tolerated. It does not require general anaesthesia and mechanical ventilation because many patients have severe co-morbidity that could make such a procedure too risky.^24^ It enables early palliative measures such as complete removal of pleural fluid, especially where mechanical intervention is required to fenestrate loculized effusion and to perform pleurodesis in incurable patients. Semirigid thoracoscopy has several potential advantages, among which the most prominent is easy and more extensive maneuverability without application of excessive force to the ribs during navigational efforts. Comprehensive examination of the pleural space enables better selection of biopsy sites, which is reflected in a high diagnostic yield. Pleurodesis with talc insufflation under direct supervision is feasible and effective. There may also be some disadvantages such as a narrow working channel and especially weak and bendable biopsy forceps, which could slow down the biopsy process and thus the entire procedure.^15^ New biopsy techniques are needed to simplify the biopsy process, and biopsy with an electrocautery knife or cryobiopsy might be among these.^25^

Semirigid thoracoscopy is an effective and safe method for diagnosing and to some extent treating pleural disorders. The method is still under development, where further improvements might be expected. However, additional studies are needed to compare rigid and semirigid thoracoscopy and establish their relation and indications for each technique. We view both techniques more as complementary than competing, but further development may provide an answer to this issue.

## Figures and Tables

**TABLE 1.** Clinical and demographic characteristics of the 111 patients that underwent semirigid thoracoscopy

**Variables**	***n* (%)**
Patients	111
Procedures	115
Median age (years)	65.0 (range 28–86)
Male	95 (86.6%)
Female	16 (14.4%)
Smoking status	
Current and previous	67 (60.4%)
Non-smoker	44 (39.6%)
Asbestos exposure	
Yes	36 (32.4%)
No	75 (67.6%)
Size of effusion	
1/3 hemithorax or less	54 (46.9%)
2/3 hemithorax	50 (43.5%)
Massive	11 (9.6%)
Side	
Left	52 (45.2%)
Right	63 (54.8%)
Performance status	
ECOG 0^*^	4 (3.6%)
ECOG 1	77 (69.4%)
ECOG 2	20 (18.0%)
ECOG 3	10 (9.0%)

* ECOG = Eastern Cooperative Oncology Group

**TABLE 2.** Final diagnosis, complications, diagnostic yield, and other features of patients undergoing semirigid thoracoscopy procedures

**Variables**	***n* (%)**
Malignant disease	73 (65.8%)
Mesothelioma	48
Secondary carcinoma	24
Lymphoma	1
Benign disease	38 (34.2%)
Non-specific pleuritis	20
Asbestos pleuritis	12
TBC pleuritis	2
Rheumatoid pleurisy	1
Hemothorax	1
Number of biopsies per patient	11.2 (±3.2)
Pleural adhesions / loculations	64 (55.7%)
Trapped lung	17 (15.3%)
Talc pleurodesis	34 (29.6%)
Median duration of chest tube drainage after procedure (days)	2 (range 0–22)
Complications	
Major	6
Minor	18
Mortality at 30 days	1
Overall diagnostic accuracy (%)	97.4%
Sensitivity for malignancy (%)	96.0%
Positive predictive value for malignancy (%)	100%
Negative predictive value for malignancy (%)	93.0%
